# A framework for value-creating learning health systems

**DOI:** 10.1186/s12961-019-0477-3

**Published:** 2019-08-09

**Authors:** Matthew Menear, Marc-André Blanchette, Olivier Demers-Payette, Denis Roy

**Affiliations:** 1 Institut national d’excellence en santé et en services sociaux (INESSS), Quebec, Canada; 2 Centre de recherche sur les soins et les services de première ligne de l’Université Laval, Landry-Poulin Pavilion, 2525 chemin de la Canardière, Quebec, QC G1J 0A4 Canada; 3 Université du Québec à Trois-Rivières, Quebec, Canada

**Keywords:** Learning health systems, Framework, Quality improvement, Health system performance, Value-based care, Canada

## Abstract

**Background:**

Interest in value-based healthcare, generally defined as providing better care at lower cost, has grown worldwide, and learning health systems (LHSs) have been proposed as a key strategy for improving value in healthcare. LHSs are emerging around the world and aim to leverage advancements in science, technology and practice to improve health system performance at lower cost. However, there remains much uncertainty around the implementation of LHSs and the distinctive features of these systems. This paper presents a conceptual framework that has been developed in Canada to support the implementation of value-creating LHSs.

**Methods:**

The framework was developed by an interdisciplinary team at the Institut national d’excellence en santé et en services sociaux (INESSS). It was informed by a scoping review of the scientific and grey literature on LHSs, regular team discussions over a 14-month period, and consultations with Canadian and international experts.

**Results:**

The framework describes four elements that characterise LHSs, namely (1) core values, (2) pillars and accelerators, (3) processes and (4) outcomes. LHSs embody certain core values, including an emphasis on participatory leadership, inclusiveness, scientific rigour and person-centredness. In addition, values such as equity and solidarity should also guide LHSs and are particularly relevant in countries like Canada. LHS pillars are the infrastructure and resources supporting the LHS, whereas accelerators are those specific structures that enable more rapid learning and improvement. For LHSs to create value, such infrastructures must not only exist within the ecosystem but also be connected and aligned with the LHSs’ strategic goals. These pillars support the execution, routinisation and acceleration of learning cycles, which are the fundamental processes of LHSs. The main outcome sought by executing learning cycles is the creation of value, which we define as the striking of a more optimal balance of impacts on patient and provider experience, population health and health system costs.

**Conclusions:**

Our framework illustrates how the distinctive structures, processes and outcomes of LHSs tie together with the aim of optimising health system performance and delivering greater value in health systems.

**Electronic supplementary material:**

The online version of this article (10.1186/s12961-019-0477-3) contains supplementary material, which is available to authorized users.

## Introduction

Among the most fundamental shifts currently taking place in the healthcare landscape is the movement towards value-based healthcare [1]. Value-based healthcare strives to achieve the best possible health outcomes [1, 2] or the best possible care [3] at the lowest cost. While details surrounding the concept’s operationalisation remain debated [4, 5], the general goal of value improvement has been embraced in a growing number of healthcare settings. The concept is appealing because it represents a clear strategic focus that can potentially align the interests of all system actors and guide collective efforts to enhance healthcare system performance [2, 6]. The pursuit of greater value in health is not without its challenges, however, as it will demand significant changes to the way health systems currently deliver and fund care, share data, support innovation, and evaluate performance [6, 7].

The United States Institute of Medicine (now the National Academy of Medicine (NAM)) proposed that a key pathway towards value-based healthcare is through the implementation of learning healthcare systems (LHSs) [8, 9]. LHSs were defined as systems where “*science, informatics, incentives, and culture are aligned for continuous improvement and innovation, with best practices seamlessly embedded in the delivery process and new knowledge captured as an integral by-product of the delivery experience*” [8]. Such systems would capitalise on major technological advances in order to foster more dynamic approaches to evidence generation and application, enable rapid learning and improvement based on data flowing from routine patient care, and ensure greater quality, safety and innovation in healthcare. However, while the concept of LHSs has generated considerable enthusiasm across the United States and increasingly in other parts of the world [10–12], its implementation on a broad scale remains limited. Furthermore, LHSs that have been implemented to date vary widely in their goals, structure and scale, and have pursued different interpretations of the NAM’s views on the LHS [11, 13].

In Canada, several leading organisations now identify the delivery of high-value healthcare as an important system goal [14–18]. Similarly, recent reports have described the LHS approach as a fundamental strategy for enabling evidence-driven health system transformation across the country [19–21]. However, there remains no consensus in Canada around how value-based healthcare should be defined and little clarity about how LHSs can contribute to value improvement. The LHS concept has emerged primarily within the complex, largely privately funded United States healthcare context, which differs significantly from other systems internationally. Canada’s universal public healthcare system features different institutions, regulations and guiding values, and has historically emphasised the importance of population health approaches for social progress [22, 23]. Given these contextual differences, there is a need to clarify how the LHS concept applies in Canada and other jurisdictions sharing similar health systems characteristics.

Achieving this understanding is considered critical within the Institut national d’excellence en santé et en services sociaux (INESSS), a public organisation whose mission is to promote clinical excellence and the efficient use of resources in the health and social services sector in the province of Quebec, Canada. INESSS has been an early adopter of the notions of value and learning systems [16, 24] and has recently engaged with other Canadian partners to establish a shared vision for the future. The purpose of this paper is to present the conceptual framework that emerged from these efforts to guide progress towards value-creating LHSs.

## Methods

The process we used to develop our conceptual framework included (1) a scoping review to identify definitions, models and case examples of LHSs, (2) interdisciplinary expert discussion meetings to build the conceptual framework, and (3) consultations with experts. The scoping review involved iterative searches in publication databases, journals and the grey literature [25]. Database searches were conducted in Medline, Embase and the Cochrane Library using keywords such as “learning healthcare system” and “learning health system”. We also performed searches for articles in the journal *Learning Health Systems*. Finally, we performed similar keyword searches in Google and consulted the websites of organisations or groups with a clear interest in learning systems (e.g. NAM, Academy Health, Agency for Healthcare Research and Quality, The Learning Healthcare Project). There were no restrictions on the types of articles or reports eligible for consideration, as long as they had LHSs as a primary focus. Among the over 80 articles we retrieved, 18 provided conceptual descriptions or frameworks for the LHS. We also identified 37 case examples of LHSs whose goals, structures and processes were sufficiently described to inform our framework (Additional file 1).

A team of four experts from INESSS with different backgrounds (e.g. health management, public health, epidemiology) met regularly between September 2017 and November 2018 to develop the conceptual framework. The development process was iterative and involved frequent discussions of cases and frameworks identified through the scoping review and concepts drawn from other seminal texts from the fields of organisational and educational sciences. Preliminary versions of the framework were presented at two Canadian health services research conferences and were revised based on participant feedback. Finally, we consulted with Canadian and international experts in health system learning and performance, including an interdisciplinary group of researchers from the University of Montreal, who provided suggestions that contributed to a final version of the framework and shared their perspectives on implementation issues in Canadian contexts.

## Results

### Other LHS conceptual frameworks

The scoping review helped us identify articles and reports providing helpful conceptual clarifications of the LHS or its components. An example is the strategy map for value and science-driven healthcare developed by leading healthcare experts in the United States [8, 9]. This strategy map outlines a vision for LHSs and their foundational elements (e.g. clinical data, information technology, evidence standards, patient engagement), characteristics (e.g. care-driven learning, person-centred, networked leadership), collaborative actions (e.g. clinical effectiveness research, best practices, value incentives) and goals (i.e. value based on health outcomes and costs). Whereas the map offers a comprehensive and high-level depiction of LHS strategic objectives, insights into its operationalisation can only be gleaned by consulting multiple NAM reports [8].

Friedman et al. [10, 26] have proposed a compelling framework that depicts the basic learning cycle at the heart of LHSs and the infrastructures that support the execution of these cycles. Learning cycles can occur at various speeds and levels of scale but invariably consist of three core processes, namely (1) converting data to knowledge (D2K), (2) applying knowledge to influence performance (K2P), and (3) documenting changes in performance to generate new data (P2D). Technologies, policies and standards constitute the main infrastructures that support these cycles and the LHS. Similar to the NAM, Friedman et al. [10] also identify improving health and reducing costs and other harms as the primary goals of LHSs. Other authors have provided alternative views on the LHS learning cycle, either in the number of cycle stages or their supporting infrastructures [13, 27–29].

Two frameworks that pay particular attention to LHS infrastructures and activities are the frameworks of Psek et al. [30] and Lessard et al. [13]. Psek et al. [30] describe the framework used within Geisinger Health System, which comprises nine components, as follows: data and analytics, people and partnerships, patient and family engagement, ethics and oversight, evaluation and methodology, funding, organisation, prioritisation, and deliverables. For their part, Lessard et al. [13] recently proposed an LHS architectural framework, or high-level blueprint, intended to guide health system actors in decisions about the design and implementation of context-adapted LHSs. Their framework comprises six decision layers that reflect common LHS dimensions identified in the literature, namely (1) the performance layer (e.g. strategic goals pursued by the LHS), (2) the scientific layer (e.g. the learning cycles and activities undertaken in the LHS), (3) the organisational layer (e.g. governance models and organisational structures), (4) the data layer (e.g. the ways data is collected, used and shared), (5) the information technology (IT) layer (e.g. the IT systems in place), and (6) the ethics and security layer (e.g. the ethical and privacy measures adopted). In addition to these more global frameworks are efforts to conceptualise specific infrastructures within the LHS, notably its technological structures [31–36].

Each of the frameworks described above helped us establish a comprehensive view of LHSs. However, some processes related to learning and improvement remained unclear and we noted that relatively few authors drew from existing theoretical literature in developing their frameworks, including important conceptual precursors such as the works on learning organisations by Senge [37]. In some cases, it was also evident that the Canadian healthcare context necessitated different considerations with respect to the values or structures underpinning LHSs. In the sections that follow, we expand on these issues and describe the framework developed at INESSS that builds on these previous works.

### LHS definition and framework elements

**Fig. 1:**
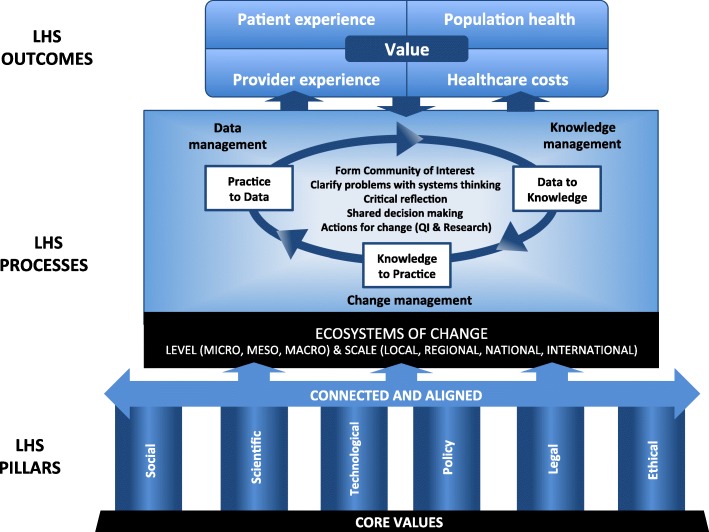
Conceptual framework for value-creating learning health systems

As noted recently by Lavis et al. [21], the term ‘learning health system’ is preferable to ‘learning healthcare system’ in Canadian contexts given the language commonly used in this country and our emphasis on other determinants of health outside of the realm of healthcare. We thus define LHSs as dynamic health ecosystems where scientific, social, technological, policy, legal and ethical dimensions are synergistically aligned to enable cycles of continuous learning and improvement to be routinised and embedded across the system, thus enhancing value through an optimised balance of impacts on patient and provider experience, population health and health system costs. In line with population health approaches, we see LHSs influencing and being influenced by the complex environments in which they evolve. These ecosystems of change can be characterised by their level and scale. Three levels can be defined – (1) the microsystem level, which features the organisations and units that produce health and social services; (2) the mesosystem level, where service continuums and joint action programmes are defined; and (3) the macrosystem level, where decisions about population-level planning and overall system performance occur [38]. Similarly, LHSs can vary in their scale, operating locally, regionally, nationally or internationally. This conceptualisation implies that local or regional LHSs can evolve alongside or within broader LHSs, with linkages between LHSs or between actors at various system levels. The framework for value-creating LHSs is depicted in Fig. 1 and consists of four elements, namely (1) core values, (2) pillars and accelerators, (3) processes, and (4) outcomes.

### Core values

Core values that should underpin LHSs have been identified previously and include accessibility, adaptability, cooperative and participatory leadership, governance, inclusiveness, person focused, privacy, scientific integrity, transparency, and value in healthcare [39]. However, in Canada and other countries, implementation of LHSs may be challenged if these systems disregard other core societal values or health system objectives such as equity, fairness and solidarity [40–42]. These values can be reflected by meaningful efforts to engage diverse stakeholders in the LHS, empower groups with marginalised voices, promote a sense of collective responsibility for its activities and outcomes, and ensure that its impacts are fairly distributed and serve to reduce disparities in care experiences or population health [43]. Additionally, given the plural leadership in the Canadian health sector, spread among governments, professional groups and actors from other sectors, as well as the growing expectations around public input into healthcare [40], we refer to the value of ‘shared accountability’ rather than ‘governance’. Canadians further expect their health system to remain responsive to their evolving needs through innovation and collaboration [19, 44]. The value of ‘open innovation’ reflects this more collaborative approach to innovation that LHSs should strive to achieve [45]. Finally, we viewed ‘value in healthcare’ not as an underpinning value but rather as the driving motive for LHSs. Our list of core LHS values with their definitions appears in Table 1.

**Table 1:** Learning health system (LHS) core values

Core value	Definition
Adaptability	The LHS will be designed to enable iterative, rapid adaptation and incremental evolution to meet the current and future needs of stakeholders
Cooperative and participatory leadership	The leadership of the LHS will be a multi-stakeholder collaboration that empowers stakeholders to participate meaningfully in LHS decisions and activities
Equity	The LHS examines problems and solutions with an equity lens and aims to ensure that its impacts are fairly distributed and reduce population health disparities
Inclusiveness	Every individual and organisation committed to the goals of the LHS is invited and encouraged to participate
Open innovation	LHS leverages knowledge from multiple internal and external sources and promotes collaborative approaches to innovation and the flow of ideas across organisational boundaries.
Person focused	The LHS will engage patients, families, communities and the general public as partners in its governance and activities, focus on their priorities, and strive to improve outcomes at individual patient and population levels that matter to them
Privacy	The LHS will protect the privacy, confidentiality and security of all data to enable responsible sharing of data, information and knowledge
Scientific integrity	The LHS and its participants will share a commitment to the most rigorous application of science to ensure the validity and credibility of findings
Shared accountability	A well-designed system of governance will allow stakeholders to share accountability for LHS strategies, policies, standards and outcomes
Solidarity	The LHS unites stakeholders with a common interest, empowers those with marginalised voices, builds trust between members, and promotes a collective responsibility for delivering value to all members and the public
Transparency	All aspects of the LHS will be open and transparent to safeguard and deepen the trust of all stakeholders

### LHS pillars and accelerators

LHS pillars refer to the infrastructure, systems and resources that provide the foundational supports for LHSs, whereas accelerators represent specific supports that accelerate learning within these systems (i.e. create ‘rapid’ LHSs). We identified six main pillars – scientific, social, technological, policy, legal and ethical pillars, though some LHS supports could align with multiple pillars at the same time. Table 2 presents the elements within these pillars and examples drawn from the 37 LHS cases identified. Importantly, each of these pillars provides necessary but not sufficient elements needed to support the LHS. Indeed, what matters less is the presence of infrastructure and supports within an ecosystem, and more the synergy between these elements and how they can be connected or aligned to achieve LHS goals [21].

**Table 2:** Learning health system pillars and accelerators

Pillar	Elements	Examples of accelerators
Scientific	• Scientific expertise • Academic or research institutes, centres, and groups • Research training programmes and knowledge-sharing activities • Research funding agencies and programmes	• Kaiser Permanente Learning Health System Program for research that drives continuous learning and improvement [46] • Armstrong Institute for Patient Safety and Quality, a transdisciplinary group that coordinates research, training and quality improvement across the Johns Hopkins Medicine system [47] • NUCAT’s Center for Data Science and Informatics [48] • CATALyst Scholar Program at Kaiser Permanente Washington Health Research Institute [49] • Funding programmes for research on new delivery models and patient-centred outcomes from the Agency for Healthcare Research and Quality and the PCORI [50]
Social	• Multi-stakeholder networks and learning communities • Service or partnership agreements • Stakeholder engagement mechanisms (e.g. committees, advisory groups)	• Multidisciplinary teams and working groups within strategic clinical networks in Alberta [51] • ‘Clinical communities’ bringing together clinicians and researchers at Johns Hopkins Medicine [47] • DARTNet learning communities enabling learning from high performing clinical sites [52] • Geisinger Health System Patient and Family Advisory Council and Patient Experience Steering Committee [30] • ImproveCareNow Exchange online knowledge and resource hub [53] • Change Group within regional community of practice in lung cancer care [29]
Technological	• Expertise in information technology and data science • Information technology systems • Health technologies or devices • Data infrastructures (e.g. electronic health records, clinical or administrative databases, clinical registry) • Communication technologies and platforms • Web or mobile applications • Data warehouses and marts • Interoperability frameworks	• Kaiser Permanente HealthConnect electronic health records system [54] • PCORNet Distributed Research Network Architecture [55] • EHR-linked multicentre clinical registries [32, 56] • Data warehouses supporting research and clinical care [48, 57–59] • Open source tools for data access, queries and analysis [31, 58] • Dashboards for visualisation of EHR or clinical registry data [29, 34, 60] • Electronic systems for capturing patient-reported outcomes data [61, 62] • Machine learning algorithms used in CancerLinQ [63] • Listserv for communication across IBD care centres [60]
Policy	• Governance and accountability structures and systems • LHS policies • LHS performance frameworks and incentive systems • Funding mechanisms for LHS operations and sustainability	• Steering and advisory committees of the PaTH LHS [58] • Governance Councils and performance milestones within LHSNet [64] • Data collaboration agreements governing sharing and use of data across sites [59] • Accountability chain at Johns Hopkins Medicine [47] • Merit-based incentive system for EHR adoption through the MACRA [65, 66] • Data quality assessment policies and procedures [61, 63]
Legal	• Privacy legislation • Laws governing healthcare institutions, organisations and professionals • Other laws, regulations and rules relevant to LHS activities	• HITECH Act [67] • MACRA Act [66]
Ethical	• Ethics expertise • Ethical review boards and committees • Ethics guidelines, frameworks and rules	• CancerLinQ regulatory framework and guiding principles for the ethical management and use of data [63, 68] • Educational initiative for Geisinger Health system Institutional Review Board members on the ethical challenges of research and innovation within LHSs [30] • Regulatory Workgroup in LHSNet to streamline IRB processes and enable more rapid project start-up and IRB approval [64] • ‘Triple use’ registry protocol describing how registry data would be simultaneously used for chronic care management, quality improvement and research [32]

*EHR* electronic health record; *HITECH* Health Information Technology for Economic and Clinical Health; *IBD* inflammatory bowel disease; *IRB* institutional review board; *LHS* learning health system; *LHSNet* Patient-Centered Network of Learning Health Systems; *MACRA* Medicare Access and CHIP Reauthorization Act; *NUCAT* Northwestern University Clinical and Translational Sciences Institute; *PaTH* University of Pittsburgh/UPMC, Penn State College of Medicine, Temple University Hospital, and Johns Hopkins University; *PCORI* Patient-Centered Outcomes Research Institute

The scientific pillar encompasses the range of scientific infrastructure, programmes and resources that support knowledge generation, sharing and application within the LHS and promote evidence-informed healthcare. This includes academic or research institutes, centres or groups that can nurture and mobilise scientific expertise relevant to the LHS. Entities specifically dedicated to advancing the LHS model (e.g. Kaiser’s LHS programme [46]) can accelerate progress towards more rapid LHSs. This pillar also includes training programmes and educational activities that build the individual and collective expertise needed for the LHS. Competencies thought to be critical for learning systems include systems science, data science, research methods for real-world contexts, implementation science, and participatory research and quality improvement (QI) approaches [69]. Having individuals that can master several of these competencies at once and work as embedded researchers [69] or knowledge brokers [70] should also accelerate learning in these systems. Funding agencies and programmes are also key components of the scientific pillar, with more flexible, rapid cycle and applied research funding programmes serving as accelerators [71].

The social pillar includes the actors and networks that interact within the LHS and the norms, culture and partnerships that establish its unique identity. LHSs are shaped and made successful by the combined efforts of the people within them, which may include healthcare providers, administrators, policy-makers, patients, community members, researchers, industry partners or other experts. Depending on the level and scale of the ecosystem, different social arrangements and mechanisms of communication and collaboration (e.g. teams, committees, virtual communities) can be adopted to ensure a deep and active engagement of members in the LHS. Indeed, LHSs have the potential to dismantle silos that frequently exist between actors from different disciplines or organisations from different sectors [47, 72]. Another transformative feature of LHSs is their emphasis on patient and community engagement [30, 73, 74], which can take many forms, as demonstrated in large-scale initiatives such as PCORnet^®^ in the United States (https://pcornet.org) and the Canadian Institutes of Health Research’s signature SPOR (Strategy for Patient-Oriented Research) initiative in Canada [75]. Networks and learning communities that foster trusting relations between diverse stakeholders can nurture cultures in which learning and improvement is ingrained within their normal operations, though fully realising such culture shifts is considered one of the most challenging tasks of LHS implementation [76, 77].

The technological pillar is similarly central to LHSs and includes a wide range of health technologies, devices, and IT infrastructure and systems. The array of technologies relevant to the LHS has been explored by the NAM [54] but is constantly expanding and evolving. A distinctive feature of LHSs is their capacity to promote learning as a by-product of everyday care, which is only made possible through infrastructure that enables high-quality clinical data to be collected, aggregated, analysed and acted on. Some LHSs have been built around clinical registers housing uniformly collected data used to describe populations with specific diseases or characteristics and monitor their outcomes such as the registers used by ImprovingCareNow [32] or the Swedish Rheumatology Society [78]. In other LHSs, health data is gathered from electronic health record (EHR) systems (e.g. [30, 31, 36, 57, 63, 79]), patients’ personal health records (e.g. [31]) and/or research databases (e.g. [48, 80]). Data from millions of patients can be stored in centralised data warehouses where it can then be aggregated, linked with other forms of data, and then leveraged for system learning and improvement. Other platforms for integrating data based on federated or mediated approaches have also been established [35, 36, 54]. Hardware and software supporting the use of algorithms, machine learning, data mining and advances in artificial intelligence also offer unmistakable potential to identify new problems, examine trends in care, test solutions, and ultimately accelerate learning and innovation [81].

The policy pillar recognises that the goals pursued by an LHS cannot be achieved without adequate governance structures, policies, financing mechanisms and accountability measures. Leadership teams or steering committees play a pivotal role in setting the strategic directions for LHSs, establishing processes for fair and shared decision-making, negotiating roles and responsibilities, and identifying clear performance targets and deliverables that enhance value across the system. Early LHSs have introduced a variety of policies to support system governance, including policies on membership and regulating member actions, data quality, data ownership and use, security and privacy, and quality assurance [61, 63, 82]. Policies related to whether and how financial and other resources are shared and how financial sustainability of the LHS is ensured are also important elements of this pillar. Incentive systems and performance frameworks that foster greater alignment with LHS structures and processes (e.g. incentives for implementing EHR systems, value-based funding models) should also accelerate progress towards more rapid LHSs.

Legislators and regulators similarly have a role to play in creating the conditions required for effective LHSs. The legal pillar thus includes the laws, rules and regulations that guide or constrain the LHS and govern the conduct of its members. One of the most frequently discussed areas of legislation in the LHS literature is privacy legislation. For instance, in the United States, the Health Insurance Portability and Accountability Act (HIPAA) includes provisions around privacy that introduce safeguards to protect personal health information and define the procedures and conditions under which such information can be accessed or shared by different stakeholders [83]. While such legislation is essential for public trust in the LHS, it has also at times slowed or impeded research and improvement activities [73]. Numerous other laws and regulations, such as those that define institutional mandates, regulate relations between organisations and govern professional practice, may facilitate or limit progress towards learning systems.

Finally, the ethical pillar includes the ethical structures, frameworks and guidelines that support the LHS, notably by helping stakeholders manage data ethically and navigate the blurred boundaries between clinical practice, QI, research and innovation. Current ethical frameworks, which emphasise sharp distinctions between QI and research, and burdensome oversight for the latter, can significantly hinder the real-time learning and improvement processes that characterise the LHS [84]. Training for members of institutional and ethical review boards and ethical guidelines for data stewardship and protection are strategies needed to promote rapid learning cycles [30, 63]. Promising guidance on these issues has already been produced by Faden et al. [84].

### LHS processes

Consistent with the work of Friedman [10, 85], we see the execution and routinisation of learning cycles as the fundamental processes of LHSs. Learning cycles have three phases – P2D, D2K and K2P [85]. The execution of these phases is driven by ‘communities of interest’, which comprise the core group of actors motivated to tackle a collective problem and align LHS pillars to achieve their goals. The speed at which communities of interest can execute whole learning cycles depends on various factors, including the nature of the problems and activities pursued as well as communities’ capacity to build or connect pillars and introduce accelerators. As LHS members gain experience with learning cycles, they can use technological, policy and other levers to routinise key processes within each phase, thus promoting more seamless transitions between phases and more efficient and impactful cycles overall.

Each learning cycle phase features its own set of distinct processes as well as a central management challenge. The P2D phase centres on the generation of practice-based data by providers, administrators, patients or other LHS actors collected through a variety of mechanisms. The central challenge of this phase is thus the management of this data. This includes the processes of designing and managing a data infrastructure, promoting good data governance, monitoring and ensuring data quality and security, facilitating data integration and interoperability, and establishing clear strategies for data access, storage and sharing. While IT experts, data scientists and researchers have a clear role here, the involvement of other stakeholders (e.g. clinicians, patients) is also essential for good data management. Participatory, user-centred design approaches can be used to help design data systems and processes that are less disruptive and more user-friendly and responsive to the needs of all LHS members [86].

The D2K phase of the learning cycle involves the conversion of data generated from routine care, research or QI to knowledge that can drive decision-making, improvement and innovation in the LHS. Knowledge management is thus the central challenge of this phase. From a knowledge management perspective, distinctions can be drawn between data (e.g. facts, symbols, statistics, signals), information (data given meaning, relevance or context) and knowledge (personalised information that individuals believe to be true) [87, 88]. Within the LHS, knowledge creation and learning depend on members’ ability to efficiently convert data to knowledge and subsequently use this knowledge to guide new data collection. This process is inherently social and iterative. It involves steps such as data analysis, synthesis, visualisation or verbalisation as well as protected time to collectively interpret data and establish shared meaning through sensemaking [89, 90]. This process may draw not only on explicit, codified forms of knowledge but also tacit knowledge rooted in members’ experiences. Indeed, models of organisational learning highlight the important interplay between tacit and explicit knowledge that occurs when individuals practice in their field, interact and engage in dialogue with others, and work to link their knowledge [89, 91].

In the K2P phase, the knowledge residing with communities of interest is applied to support practice innovation and improvement and ultimately deliver greater value. Managing change is thus the central challenge of this phase. The literature on change management, QI and the implementation of innovations is vast and ever expanding, spanning numerous disciplines. Points of convergence regarding key K2P processes includes processes related to making decisions about the nature of change, establishing a shared vision for change, and selecting change or implementation models or theories [92–95]. LHS leaders can prepare for change, such as by consulting partners and assessing readiness for change, identifying and minimising barriers to change, mobilising key stakeholders, soliciting the help of champions or change agents, and establishing a sense of urgency for change [94, 96, 97]. Taking action and implementing change involves the identification of new interventions or best practices that can be expected to add value, selecting from a wide range of change strategies, and potentially tailoring change strategies to particular contexts or target populations [98–101]. Communities of interest may endorse large transformations or prefer piloting innovations on a small scale or support rapid cycles of change in practice with an emphasis on quick, visible successes that can be communicated and celebrated [94, 95]. Finally, plans to institutionalise, sustain and scale-up change must be carefully considered. These plans should include strategies for monitoring change and evaluating its impacts, mobilising new partners and resources, rewarding new practices, and fostering an environment conducive to continuous learning and improvement [94–97, 102]. While such K2P processes may initially be conducted within more formal or planned QI initiatives or action research activities, LHSs should strive to achieve change processes that are increasingly emergent and continuous over time [10].

In addition to the processes linked to each learning cycle phase, several core processes are necessary in order for the LHS to achieve its ultimate aim, i.e. creating and improving value. In particular, the key actors making up a community of interest must be identified and given the opportunity to build a shared purpose and approach to working together. Next, these communities should establish robust processes for identifying, clarifying and eventually revisiting the specific problems that their members wish to address. Especially important is the need for systems thinking at this stage, including consideration of the interdependencies between members and the ‘big picture’ problems that require long-term solutions [37]. This is facilitated by critical reflection, one of the foundations for collective action, learning and improvement [103]. In contexts where time is often a scarce resource, LHS members must routinely protect time for group-based self-reflection where current practices, assumptions and solutions can be critically examined. Such reflection sets the stage for shared decision-making, in which partners work together to outline alternative courses of action, weigh their pros and cons, and make fair decisions given members’ preferences and priorities. The last core process relates to taking actions and ‘learning from doing’. The impacts of these actions should thus be monitored and reflected upon, as should the processes LHS members used to arrive at decisions and impacts, consistent with the idea of ‘double loop learning’ [104].

### LHS outcomes

At INESSS, the *raison d’être* of learning cycles and LHSs is seen as the improvement of value for people. A critical task for communities of interest is thus to achieve an accepted definition of ‘value’ and track its improvement. While Porter’s views on value [2, 6] have shifted conversations on health system performance, they have not gone unchallenged and new ideas about how to define and operationalise the concept in health contexts have been proposed [105–107]. Informed by this debate, we argue that value is created when a more optimal and acceptable balance is achieved across four dimensions of health system performance, namely patients’ care experience, providers’ care experience, population health, and health system costs [108]. LHS members are thus encouraged to define, in operational terms, what progress in the pursuit of this quadruple aim looks like. To this end, we submit three propositions intended to support this challenging task.

First, value as a concept is both complex and relative, and as such its appreciation demands that multiple complimentary perspectives be considered. LHSs will often have diffuse power structures, with members having a myriad of legitimate interests. Reconciling these interests and identifying acceptable trade-offs in health system objectives is not always easy; however, fair and informed decisions can be achieved through participatory approaches and deliberative processes that mobilise the best available evidence alongside members’ diverse experiential and contextual knowledge. The inclusion of perspectives from patients and the public is particularly central and reflected in a growing movement towards the routine collection of patient-reported outcomes and experience measures within LHSs [30, 47, 58, 61, 62, 109]. LHSs should adopt transparent methods to allow these and other stakeholders in the LHS to identify the goals most meaningful to them, considering that some goals (e.g. improving population health) are highly valued in contexts like Canada. Ultimately, LHSs may identify multiple value-related indicators covering different dimensions of LHS performance that reflect both the shared and unique interests of its members [5].

A second proposition is that it is imperative to assess value in real-world contexts and throughout the life cycle of the innovations introduced to improve health and care. Innovations such as new clinical practices, programmes or technologies can offer great promise of improved value but the extent to which this promise is fully realised should be evaluated in diverse populations and care settings. LHSs can offer an ideal environment to examine whether innovations are reaching the right people in the right way at the right time, and whether this results in better care experiences and outcomes. Communities of interest should adopt long-lasting monitoring processes to ensure that value is demonstrated and maintained after taking actions assumed to be beneficial.

Third, a holistic appraisal of value requires a deep understanding of the trajectories of care experienced by different clinical populations. As Porter argues [2], value measurements should encompass the full range of services and activities that jointly determine success in meeting patients’ needs. Indeed, from a patient’s perspective, perceptions of value will often depend less on their experiences with a single intervention or provider but rather on an accumulation of experiences as they journey through multiple contacts with the health system. Poor continuity or integration of care along this journey reduces value through negative impacts on both individual care experiences and population health. LHSs focused on improving value should characterise the key stages and services within care trajectories for specific populations and then work with those populations and care providers to determine how value can be added at different points along those trajectories. Furthermore, as LHSs make progress in the integration and interoperability of their information systems, so too will progress be made in their ability to situate patients within a service continuum and use real-time health and cost data to seize opportunities for value improvement.

## Discussion

With the introduction of the LHS concept, the NAM has provided a compelling vision for the optimisation of health systems worldwide. In this article, we describe a conceptual framework that is guiding work by INESSS to foster greater learning and improvement in Canada. This framework comprises four main components, notably LHS core values, pillars and accelerators, processes, and outcomes. We argue that LHSs serve primarily to improve value in health systems, based on definitions of value that account for the diverse interests and objectives of actors within communities of interest.

In Canada, recent reports have drawn attention to the LHS as a foundation for a higher performing healthcare system [19–21]. Still, there are many challenges that must be overcome to achieve this vision, notably at the level of LHS pillars. For example, Canada has made massive investments in its data infrastructure over the past decade, including substantial efforts to support EHR system adoption in primary care, yet this infrastructure remains plagued by problems of interoperability and the inability to link and aggregate data [21, 110–112]. Efforts to build national clinical registries or EHR-based research networks have also been slowed due to the multitude of provincial laws governing personal health information and their interpretation by ethics review boards [113, 114]. National funding for health research is not yet adapted to support rapid innovation cycles and only scant funding has been directed to change management and the scale and spread of innovations [71, 115]. Few mechanisms have been introduced to ensure strong patient involvement in health system design and priority-setting and the routine collection of patient-reported outcome measures and patient-reported experience measures is far from a reality in most health jurisdictions [21, 116]. With an inadequate socio-technical architecture, provincial health systems have been largely unable to establish a culture of learning and improvement and make progress towards the objectives of the quadruple aim [19, 21, 117, 118].

Despite these challenges, Canada still seems poised to make progress in LHS implementation over the next decade. The country features numerous prominent public agencies focused on health information and healthcare improvement whose missions seem well aligned with the notion of value-creating LHSs. INESSS has assumed a leadership role in mobilising support for these concepts, notably through its involvement in the Canadian Health Services and Policy Research Alliance (CHSPRA), which fosters collaboration, coordination and strategic investment among over 40 organisations funding and supporting health services and policy research [115]. In 2017, CHSPRA launched a training programme targeting doctoral and postdoctoral trainees that promotes embedded research within health agencies across the country. In this Health System Impact Fellowship programme, trainees develop an expanded set of competencies specifically intended to help them become scientific leaders within emerging LHSs [115]. Three authors of this paper were Health System Impact Fellows and our conceptual framework is currently being used to inform the evaluation of this pan-Canadian training programme. A CHSPRA LHS Working Group will also be using the framework to guide strategic planning of the implementation of LHSs in Canada, along with information gathered from its recently commissioned environmental scan on rapid-learning LHSs [21]. At a provincial level, our framework is informing INESSS initiatives such as the CoMPAS+ programme, which uses QI collaboratives to promote a culture of continuous QI for chronic disease prevention and management in Quebec primary care settings [119]. Already, several communities of interest have been formed to promote reflective practices around care for diabetes, chronic obstructive pulmonary disease and mental health in order to take actions to improve quality and value in primary care.

### Novel contributions

Our conceptual framework presents several strengths relative to other frameworks available in the literature. Other frameworks either do not make clear distinctions between the structural, process and outcome components of LHSs [30] or emphasise a single component (e.g. processes or structures) over others (e.g. [27, 31, 32]). Our framework is more comprehensive and illustrates, in a novel way, how communities of interest can work to align the structures, processes and outcomes that are distinct to LHSs. Moreover, our framework explicitly identifies value creation and improvement as the fundamental goal to be pursued by these communities of interest. Our conceptualisation of value based on the quadruple aim is also substantively different than those proposed by Porter and others. We note that our framework was primarily intended to meet the needs of stakeholders within the Canadian health system. This is reflected in the core values that should underpin LHSs established in Canada, the use of language that recognises multiple determinants of health, and the importance of pursuing processes and outcomes in line with population health approaches. Finally, the research conducted to develop the framework led to the identification of 37 case examples of LHSs, a more comprehensive list than what has been found in other reviews and scans [11, 21].

### Limitations

While our framework has its strengths, some limitations in our methods should be noted. First, our scoping review relied on structured searches of the literature but was not as exhaustive as a systematic review, making it possible that some articles or case examples were missed. In some countries, networks or systems may pursue similar processes or goals as in LHSs but not refer to themselves as learning systems. It would have been ideal to contact more key informants from different parts of Canada and other countries to gather feedback on the framework’s elements and identify additional case examples. How the framework may resonate in other contexts will be a focus of future work.

## Conclusion

Health systems worldwide face numerous challenges with respect to performance, rising costs and the need to more rapidly integrate evidence and innovations. The LHS concept offers much promise to help these systems benefit from scientific and technological advancements in order to optimise learning and improvement. The conceptual framework for LHSs developed at INESSS is comprehensive and defines how the distinctive components of these health systems can work together in order to achieve a shared strategic goal – the creation and improvement of value for people. The framework is being used by INESSS to guide transformation in Quebec and Canada, and may provide a useful template for health system leaders internationally.

## Additional file

Additional file 1: Learning health system case examples. (DOCX 200 kb)

## Data Availability

Data sharing is not applicable to this article as no datasets were generated or analysed during the current study.

## Abbreviations

CHSPRA: Canadian Health Services and Policy Research Alliance
D2K: data to knowledge
EHR: electronic health record
INESSS: Institut national d’excellence en santé et en services sociaux
IT: information technology
K2P: knowledge to practice
LHS: learning health system
NAM: National Academy of Medicine
P2D: practice to data
QI: quality improvement
