# Supplementary material for: A framework for value-creating learning health systems
Source: Health Res Policy Syst. 2019 Aug 9;17:79. doi: 10.1186/s12961-019-0477-3 (PMC6688264; doi:10.1186/s12961-019-0477-3)
Supplement: Supplementary file 1 — Learning health system case examples. (DOCX 200 kb) [file 12961_2019_477_MOESM1_ESM.docx]

**Additional File 1. Learning health system case examples**

| **LHS name** | **Country** | **Scale** | **Purpose** |
| --- | --- | --- | --- |
| Alberta Strategic Clinical Networks (1, 2) | Canada | Regional | Support clinically-led, evidence-informed change in pursuit of the triple aim in Alberta’s health system. |
| Athena Breast Health Network (3, 4) | United States | Regional | To integrate clinical care and research to drive innovation in patient-centered prevention, screening, treatment, and management of breast cancer. |
| CancerLinQ (5-8) | United States | National | Empower the oncology community to improve the quality of care and patient outcomes through transformational data analytics, based on a big-data, health IT platform implemented in oncology practices across the U.S. |
| Comparative Effectiveness Research Translation Network (CERTAIN) (9-11) | United States | Regional | To advance quality improvement efforts and leverage clinical registries to build a research network to design and conduct stakeholder-informed comparative effectiveness research. |
| CHEO Inflammatory Bowel Disease Centre LHS (12) | Canada | Local | To identify biomarkers for inflammatory bowel disease diagnosis and prognosis, and develop point-of-care diagnostic tools and personalized therapeutic approaches using innovative therapies. |
| Clinical Information Network (13) | Kenya | National | Improve the adoption of best practices in inpatient pediatric care in county hospitals. |
| Cystic Fibrosis Foundation Patient Registry (14) | United States | National | To describe the cystic fibrosis population in the U.S., support epidemiological and clinical research on cystic fibrosis, and improve the quality of cystic fibrosis care. |
| Dartmouth Spine Center Clinic Register (15) | United States | Local | To “feed forward” built-in data and feedback features to facilitate regular and frequent periods of physician and staff reflection, enhancing their capacity to improve health outcomes and continuously improve spine-related health outcomes. |
| Distributed Ambulatory Research in Therapeutics Network (DARTNet) (16, 17) | United States | National | To transform multi-sourced data into standardized, actionable health information that supports patient care, quality improvement, patient safety and health improvement, and collaborative learning and research. |
| EHR4CR (18) | France, UK, Germany, Poland, SW | International | Ease and speed the conduct of comparative effectiveness clinical trials using EHR data from 5 European countries. |
| ePRO cancer LHS (19) | United States | Local | To build an LHS infrastructure emphasizing patient-reported outcomes data in a cancer clinic. |
| Geisinger Health Systems LHS (20, 21) | United States | Regional | To support continuous learning and improvement across the Geisinger Health system. |
| Group Health LHS (22) | United States | Regional | To promote rapid learning and continuous improvement within the Group Health Cooperative health system. |
| Indiana University Center for Healthcare Innovation and Implementation Science (23) | United States | Regional | To use implementation science and innovation to produce great-quality, patient-centered and cost-efficient healthcare delivery solutions. |
| Inflammatory Bowel Disease – Qorus (24) | United States | National | To address evidence gaps and accelerate improvement of care for inflammatory bowel disease. |
| ImproveCareNow – Chronic Care Network (25, 26) | United States | National | To empower clinicians, researchers, parents and youth to learn and continuously improve care and outcomes for chronic diseases like inflammatory bowel disease. |
| John Hopkins Medicine LHS (27) | United States | Regional | To support continuous learning and improvement across the Johns Hopkins Medicine system. |
| Kaiser Permanente LHS (28) | United States | National | To support continuous learning and improvement across the Kaiser Permanente health systems. |
| Learn From Every Patient (LFEP) Program (29) | United States | Local | To integrate clinical care and research, and to use this knowledge to systematically deliver continual quality improvement in care. |
| LHSNet (9) | United States | National | To operationalize the learning health system across several healthcare systems. |
| NUCATS Institute LHS (30) | United States | Regional | To create a central hub supporting clinical and translational research across schools at Northwestern University. |
| Optum Labs LHS (31) | United States | National | To improve patient care and value in the healthcare system by connecting the generation of evidence with its accelerated translation into practice and its widespread adoption into care delivery. |
| Ottawa Hospital Lung Cancer LHS (32) | Canada | Regional | To drive system optimization and innovation in lung cancer care. |
| PATH LHS (33) | United States | National | To provide robust infrastructure to conduct research, explore clinical outcomes, link with biospecimens, and improve methods for sharing and analyzing data across diverse populations. |
| PEDS-CHOIR (34) | United States | Local | Tertiary care clinic registry to guide research and precision pain medicine in pediatric populations. |
| PEDSnet (35) | United States | National | To enable a community of patients and clinicians, interacting at the point of care, to generate data that can be repurposed for research and quality improvement and to support continuous monitoring of outcomes that identify specific practices as targets for comparative effectiveness research. |
| PORTAL Network (36) | United States | National | To conduct large-scale observational comparative effectiveness research and pragmatic clinical trials across diverse clinical care settings. |
| pSCANNER (37) | United States | National | To provide a secure, scalable distributed infrastructure to facilitate comparative effectiveness research and provide flexibility to participant sites in the means for data-driven collaboration. |
| SCIHLS (38) | United States | National | To support comparative effectiveness research at a national scale. |
| South Carolina Data Warehouse (39) | United States | Regional | To accelerate research to improve health through a novel collaborative mode that would maximize the value of constrained resources for the greatest benefit. |
| Swedish Rheumatology Quality Register (15, 40) | Sweden | National | To improve the healthcare and treatment of patients with rheumatoid arthritis. |
| Swiss LHS (41) | Switzerland | National | To strengthen the health system in Switzerland and create a culture of shared decision-making in which sound scientific evidence is continuously integrated into the health system. |
| TRANSFORM (42, 43) | UK, Greece,  Netherlands, Belgium, Poland | International | To provide a digital infrastructure to support the emergence of learning health systems for primary care in 5 European countries. |
| University of Wisconsin Health LHS (44) | United States | Regional | To consistently deliver high value care and support continuous learning and improvement in the University of Wisconsin health system. |
| U.S. Food and Drug Administration Sentinel Initiative (45) | United States | National | To monitor and improve the safety FDA-regulated products, including drugs, vaccines, biologics and medical devices. |
| Virgen Del Rocio University Hospital LHS (46) | Spain | Local | To support learning and decision making for genetic testing around gynecological cancer in a large Spanish university hospital. |
| Wound Care LHS (47) | United States | National | To integrate wound care management, quality improvement, and comparative effectiveness research by harnessing structured real-world data within a purpose-built electronic health record at the point of care. |

**References**

1. Noseworthy T, Wasylak T, O'Neill B. Strategic clinical networks in Alberta: Structures, processes, and early outcomes. Healthc Manage Forum. 2015;28(6):262-4.

2. Noseworthy T, Wasylak T, O'Neill BJ. Strategic Clinical Networks: Alberta's Response to Triple Aim. Healthc Pap. 2016;15(3):49-54.

3. Elson SL, Hiatt RA, Anton-Culver H, Howell LP, Naeim A, Parker BA, et al. The Athena Breast Health Network: developing a rapid learning system in breast cancer prevention, screening, treatment, and care. Breast Cancer Res Treat. 2013;140(2):417-25.

4. Lewis S, Bloom J, Rice J, Naeim A, Shortell S. Using teams to implement personalized health care across a multi-site breast cancer network. Adv Health Care Manag. 2014;16:71-94.

5. Schilsky RL, Michels DL, Kearbey AH, Yu PP, Hudis CA. Building a rapid learning health care system for oncology: the regulatory framework of CancerLinQ. J Clin Oncol. 2014;32(22):2373-9.

6. Shah A, Stewart AK, Kolacevski A, Michels D, Miller R. Building a Rapid Learning Health Care System for Oncology: Why CancerLinQ Collects Identifiable Health Information to Achieve Its Vision. J Clin Oncol. 2016;34(7):756-63.

7. Yu PP. Perspectives on the Implementation of an Ethical Rapid Learning Health System. J Oncol Pract. 2017;13(3):151-3.

8. Rubinstein SM, Warner JL. CancerLinQ: Origins, Implementation, and Future Directions. JCO Clin Cancer Inform. 2018;2:1-7.

9. Flum DR, Alfonso-Cristancho R, Devine EB, Devlin A, Farrokhi E, Tarczy-Hornoch P, et al. Implementation of a "real-world" learning health care system: Washington State's Comparative Effectiveness Research Translation Network (CERTAIN). Surgery. 2014;155(5):860-6.

10. Devine EB, Capurro D, van Eaton E, Alfonso-Cristancho R, Devlin A, Yanez ND, et al. Preparing Electronic Clinical Data for Quality Improvement and Comparative Effectiveness Research: The SCOAP CERTAIN Automation and Validation Project. EGEMS (Wash DC). 2013;1(1):1025.

11. Devine EB, Van Eaton E, Zadworny ME, Symons R, Devlin A, Yanez D, et al. Automating Electronic Clinical Data Capture for Quality Improvement and Research: The CERTAIN Validation Project of Real World Evidence. EGEMS (Wash DC). 2018;6(1):8.

12. Chuong KH, Mack DR, Stintzi A, O'Doherty KC. Human Microbiome and Learning Healthcare Systems: Integrating Research and Precision Medicine for Inflammatory Bowel Disease. OMICS. 2018;22(2):119-26.

13. Irimu G, Ogero M, Mbevi G, Agweyu A, Akech S, Julius T, et al. Approaching quality improvement at scale: a learning health system approach in Kenya. Arch Dis Child. 2018.

14. Schechter MS, Fink AK, Homa K, Goss CH. The Cystic Fibrosis Foundation Patient Registry as a tool for use in quality improvement. BMJ Qual Saf. 2014;23 Suppl 1:i9-14.

15. Ovretveit J, Nelson E, James B. Building a learning health system using clinical registers: a non-technical introduction. J Health Organ Manag. 2016;30(7):1105-18.

16. Pace WD, Cifuentes M, Valuck RJ, Staton EW, Brandt EC, West DR. An electronic practice-based network for observational comparative effectiveness research. Ann Intern Med. 2009;151(5):338-40.

17. Pace WD, Fox CH, White T, Graham D, Schilling LM, West DR. The DARTNet Institute: Seeking a Sustainable Support Mechanism for Electronic Data Enabled Research Networks. EGEMS (Wash DC). 2014;2(2):1063.

18. Daniel C, Ouagne D, Sadou E, Paris N, Hussain S, Jaulent MC, et al. Cross border semantic interoperability for learning health systems: The EHR4CR semantic resources and services. Learning Health Systems. 2017;1:e10014.

19. Abernethy AP, Ahmad A, Zafar SY, Wheeler JL, Reese JB, Lyerly HK. Electronic patient-reported data capture as a foundation of rapid learning cancer care. Med Care. 2010;48(6 Suppl):S32-8.

20. Psek WA, Stametz RA, Bailey-Davis LD, Davis D, Darer J, Faucett WA, et al. Operationalizing the learning health care system in an integrated delivery system. EGEMS (Wash DC). 2015;3(1):1122.

21. Psek W, Davis FD, Gerrity G, Stametz R, Bailey-Davis L, Henninger D, et al. Leadership Perspectives on Operationalizing the Learning Health Care System in an Integrated Delivery System. EGEMS (Wash DC). 2016;4(3):1233.

22. Greene SM, Reid RJ, Larson EB. Implementing the learning health system: from concept to action. Ann Intern Med. 2012;157(3):207-10.

23. Azar J, Adams N, Boustani M. The Indiana University Center for Healthcare Innovation and Implementation Science: Bridging healthcare research and delivery to build a learning healthcare system. Z Evid Fortbild Qual Gesundhwes. 2015;109(2):138-43.

24. Johnson LC, Melmed GY, Nelson EC, Holthoff MM, Weaver SA, Morgan TS, et al. Fostering Collaboration Through Creation of an IBD Learning Health System. Am J Gastroenterol. 2017;112(3):406-8.

25. Marsolo K. In Search of a Data-in-Once, Electronic Health Record-Linked, Multicenter Registry-How Far We Have Come and How Far We Still Have to Go. EGEMS (Wash DC). 2013;1(1):1003.

26. Marsolo K, Margolis PA, Forrest CB, Colletti RB, Hutton JJ. A Digital Architecture for a Network-Based Learning Health System: Integrating Chronic Care Management, Quality Improvement, and Research. EGEMS (Wash DC). 2015;3(1):1168.

27. Pronovost PJ, Mathews SC, Chute CG, Rosen A. Creating a purpose-driven learning and improving health system: The Johns Hopkins Medicine quality and safety experience. Learning Health Systems. 2017;1:e10018.

28. Liu VX, Morehouse JW, Baker JM, Greene JD, Kipnis P, Escobar GJ. Data that drive: Closing the loop in the learning hospital system. J Hosp Med. 2016;11 Suppl 1:S11-S7.

29. Lowes LP, Noritz GH, Newmeyer A, Embi PJ, Yin H, Smoyer WE, et al. 'Learn From Every Patient': implementation and early results of a learning health system. Dev Med Child Neurol. 2017;59(2):183-91.

30. Starren JB, Winter AQ, Lloyd-Jones DM. Enabling a Learning Health System through a Unified Enterprise Data Warehouse: The Experience of the Northwestern University Clinical and Translational Sciences (NUCATS) Institute. Clin Transl Sci. 2015;8(4):269-71.

31. Wallace PJ, Shah ND, Dennen T, Bleicher PA, Crown WH. Optum Labs: building a novel node in the learning health care system. Health Aff (Millwood). 2014;33(7):1187-94.

32. Fung-Kee-Fung M, Maziak DE, Pantarotto JR, Smylie J, Taylor L, Timlin T, et al. Regional process redesign of lung cancer care: a learning health system pilot project. Curr Oncol. 2018;25(1):59-66.

33. Amin W, Tsui FR, Borromeo C, Chuang CH, Espino JU, Ford D, et al. PaTH: towards a learning health system in the Mid-Atlantic region. J Am Med Inform Assoc. 2014;21(4):633-6.

34. Bhandari RP, Feinstein AB, Huestis SE, Krane EJ, Dunn AL, Cohen LL, et al. Pediatric-Collaborative Health Outcomes Information Registry (Peds-CHOIR): a learning health system to guide pediatric pain research and treatment. Pain. 2016;157(9):2033-44.

35. Forrest CB, Margolis PA, Bailey LC, Marsolo K, Del Beccaro MA, Finkelstein JA, et al. PEDSnet: a National Pediatric Learning Health System. J Am Med Inform Assoc. 2014;21(4):602-6.

36. McGlynn EA, Lieu TA, Durham ML, Bauck A, Laws R, Go AS, et al. Developing a data infrastructure for a learning health system: the PORTAL network. J Am Med Inform Assoc. 2014;21(4):596-601.

37. Ohno-Machado L, Agha Z, Bell DS, Dahm L, Day ME, Doctor JN, et al. pSCANNER: patient-centered Scalable National Network for Effectiveness Research. J Am Med Inform Assoc. 2014;21(4):621-6.

38. Mandl KD, Kohane IS, McFadden D, Weber GM, Natter M, Mandel J, et al. Scalable Collaborative Infrastructure for a Learning Healthcare System (SCILHS): architecture. J Am Med Inform Assoc. 2014;21(4):615-20.

39. Turley CB, Obeid J, Larsen R, Fryar KM, Lenert L, Bjorn A, et al. Leveraging a Statewide Clinical Data Warehouse to Expand Boundaries of the Learning Health System. EGEMS (Wash DC). 2016;4(1):1245.

40. Eriksson JK, Askling J, Arkema EV. The Swedish Rheumatology Quality Register: optimisation of rheumatic disease assessments using register-enriched data. Clin Exp Rheumatol. 2014;32(5 Suppl 85):S-147-9.

41. Boes S, Mantwill S, Kaufmann C, Brach M, Bickenbach J, Rubinelli S, et al. Swiss Learning Health System: A national initiative to establish learning cycles for continuous health system improvement. Learning Health Systems. 2018;2:e10059.

42. Delaney BC, Curcin V, Andreasson A, Arvanitis TN, Bastiaens H, Corrigan D, et al. Translational Medicine and Patient Safety in Europe: TRANSFoRm--Architecture for the Learning Health System in Europe. Biomed Res Int. 2015;2015:961526.

43. Ethier JF, McGilchrist M, Barton A, Cloutier AM, Curcin V, Delaney BC, et al. The TRANSFoRm project: Experience and lessons learned regarding functional and interoperability requirements to support primary care. Learning Health Systems. 2017;e10037.

44. Kraft S, Caplan W, Trowbridge E, Davis S, Berkson S, Kamnetz S, et al. Building the learning health system: Describing an organizational structure to support continuous learning. Learning Health Systems. 2017;1:e10034.

45. Institute of Medicine. Digital infrastructure for the Learning Health System: The foundation for continuous improvement in health and health care: Workshop series summary. Washington, DC: 2011.

46. Suarez-Mejias C, Martinez-Garcia A, Martinez-Maestre MA, Silvan-Alfaro JM, Moreno Conde J, Parra-Calderon CL. Learning Healthcare System for the Prescription of Genetic Testing in the Gynecological Cancer Risk. Stud Health Technol Inform. 2017;235:96-100.

47. Serena TE, Fife CE, Eckert KA, Yaakov RA, Carter MJ. A new approach to clinical research: Integrating clinical care, quality reporting, and research using a wound care network-based learning healthcare system. Wound Repair Regen. 2017;25(3):354-65.
